# Equivalent efficacy of left versus right hemisphere accelerated intermittent theta burst stimulation for major depressive disorder

**DOI:** 10.3389/fpsyt.2026.1745388

**Published:** 2026-03-11

**Authors:** Davin K. Quinn, Joel Upston, Thomas R. Jones, Tessa A. Olmstead, Justine Yang, Samuel Reyes, Samuel MacDonald, Adam Littleton, Alexander Win, Dorothy H. Bowers-Wu, Jordan G. Lee, Pearl Huynh, Robert T. DeBurlo, Alyssa Velasco, Ali Nakip, Elizabeth R. Richardson, Justin R. Davis, Shawn Hazlewood, Crystal A. Garcia, Cesar J. Ojeda, Karen Luo, Julian David, Benjamin C. Gibson, Gregory M. Nikogosyan, Brant W. Hager, Danielle Farrar, Orrin Myers, Andrei A. Vakhtin, Christopher C. Abbott

**Affiliations:** 1 Department of Psychiatry and Behavioral Sciences, University of New Mexico School of Medicine, Albuquerque, NM, United States; 2 Department of Neurosciences, University of New Mexico, Albuquerque, NM, United States; 3 University of New Mexico School of Medicine, Albuquerque, NM, United States; 4 Department of Psychology, University of New Mexico, Albuquerque, NM, United States; 5 Department of Family and Community Medicine, University of New Mexico, Albuquerque, NM, United States; 6 Mind Research Network, Albuquerque, NM, United States

**Keywords:** accelerated intermittent theta burst stimulation, electric field distribution, functional connectivity, hemispheric asymmetry, major depression (MDD)

## Abstract

**Background:**

Intermittent theta burst stimulation (iTBS) to the dorsolateral prefrontal cortex (DLPFC) for major depression has been FDA-approved in the United States since 2018. Accelerated iTBS (aiTBS) protocols of multiple treatments per day have shown promising response and remission rates for major depression, especially when combined with connectivity-guided targeting. Brain networks associated with emotion regulation demonstrate significant changes in connectivity after effective iTBS. However, these findings have been confined to treatment of the left DLPFC, despite literature suggesting equivalent outcomes with right side stimulation. To date there has not been a direct comparison of clinical outcomes and connectivity changes between left and right DLPFC aiTBS for depression.

**Methods:**

Forty-four patients aged 50–79 with chronic major depressive disorder underwent open-label accelerated fMRI-guided aiTBS (45 sessions, 9 days) to the DLPFC (18 left, 26 right). Depression, anxiety, and anhedonia symptoms were assessed, and resting-state fMRI was obtained at baseline (Visit 1), after 15 sessions (Visit 2), and end of treatment (Visit 3). Patients who were not demonstrating at least 10% improvement in depression at Visit 2 were switched to contralateral stimulation for the remaining 30 sessions.

**Results:**

For the entire cohort (N = 44), mean depression (IDS-C_30_) scores decreased significantly over the course of treatment. Mean change in IDS-C_30_, GAD-7, TEPS, SHAPS, and BISBAS between participants with right-sided stimulation (N = 26) and those with left-sided stimulation (N = 18) were not statistically different. Functional connectivity analysis demonstrated significant decreases in connectivity from Visit 1 to Visit 3 between default mode network and limbic networks in patients receiving right DLPFC iTBS, whereas patients receiving left DLPFC iTBS demonstrated limited changes in connectivity.

**Conclusion:**

Accelerated iTBS to the right DLPFC appears to have equivalent efficacy as aiTBS to the left DLPFC in terms of magnitude of reduction of depressive, anxious, and anhedonic symptoms in a late-life population. However, connectivity changes associated with treatment were asymmetric, and may reflect hemispheric lateralization of functional network responses to iTBS. Further work is needed to confirm the comparative efficacy and network dynamics of left versus right hemisphere aiTBS for depression.

## 1 Introduction

Since the first demonstrations of antidepressant effects of repetitive transcranial magnetic stimulation (rTMS) in the 1990s (1, 2), the theory of hemispheric lateralization of emotion regulation in the prefrontal cortex has guided parameter selection. Excitatory 10Hz rTMS to the left dorsolateral prefrontal cortex (DLPFC) was found by George et al. and Pascual-Leone et al. to improve depressive symptoms (2, 3), while 10Hz stimulation to the right DLPFC did not (2), suggesting a specific therapeutic effect of left hemisphere excitation. Larger controlled studies of 10Hz left DLPFC rTMS confirmed the efficacy of this paradigm (4, 5), while a handful of studies of 10Hz right DLPFC rTMS carried out yielded only mixed results (6, 7). Inhibitory paradigms such as 1Hz rTMS to the right DLPFC were subsequently found to have equivalent or greater efficacy as 10Hz rTMS to the left DLPFC, supporting a conceptualization of the left and right hemispheres as having opposite and antagonistic roles in regulating emotion (8–10).

FDA approval following pivotal trial results of left 10Hz rTMS established this approach as the dominant therapeutic paradigm (5), with 1Hz right DLPFC rTMS used as an off-label adjunct in bilateral treatments to boost antidepressant effect and reduce anxiety (11, 12). This general approach to “activate” or facilitate the left prefrontal cortex and “deactivate” or inhibit the right prefrontal cortex continues to be a guiding principle for rTMS clinicians, and has been extended with more efficient stimulation patterns such as intermittent theta burst stimulation (iTBS) for excitatory modulation for the left DLPFC and continuous theta burst stimulation (cTBS) for inhibition of the right DLPFC (13, 14).

In spite of the volume of studies affirming left excitatory/right inhibitory lateralization and its widespread adoption in clinical settings, a smaller body of mechanistic and empirical evidence has accumulated over the last decade challenging its underlying premises. Initially, the left hemisphere was theorized to be associated with positively-valenced emotions and approach behavior, while the right hemisphere was associated with negatively-valenced emotions and withdrawal behavior (15, 16). Early lesion studies demonstrated increased rates of depression after left hemisphere stroke compared to strokes in the right hemisphere (17), and a smaller corollary effect of increased mania after right hemisphere lesions (18). However, larger contemporary studies of predictors of poststroke depression in the 21^st^ century have not confirmed consistent lateralization of emotion regulation, and found depression to be prevalent in strokes to either hemisphere (19–21).

Similarly, early functional imaging studies examining cerebral blood flow, metabolic activity, and neuronal activation in healthy and clinically depressed populations provided initial evidence for hemispheric asymmetry in mood control (22–24). For instance, hypometabolism in the left frontal lobe was observed in depressed patients using positron emission tomography (PET) (25, 26), with subsequent increase in cerebral metabolism associated with antidepressant effect of 10Hz rTMS to the left DLPFC (27). Reduced left frontal cerebral blood flow on single-positron emission computed tomography (SPECT) (28) and greater left frontal alpha frequency band power measured with resting-state electroencephalography (EEG) were also found to be associated with depression (29). However, as more follow-up studies and meta-analyses investigated these findings, the effect sizes of lateralized differences have generally been attenuated (7, 30).

In the clinical arena, there has been parallel evidence from recent rTMS treatment studies demonstrating significant antidepressant effects with excitatory stimulation to the right DLPFC. In several open label studies, robust antidepressant effects were observed in patients undergoing right DLPFC iTBS (31, 32). In small controlled studies of rTMS for anxiety (33), OCD (34), and PTSD (35) symptoms, depression symptoms were measured as secondary outcomes and found to be significantly improved. These clinical findings are supported by multimodal imaging analyses performed by Siddiqi et al, in which magnetic resonance data in patients with stroke lesions or receiving deep brain stimulation or rTMS for depression were combined to produce heatmaps of brain regions associated with depression and antidepressant response (36). The symmetry of these network maps suggest that identical rTMS treatment parameters to either hemisphere may produce similar behavioral results.

In light of this reconsideration of the lateralization of emotion regulation, we sought to compare the efficacy of accelerated image-guided iTBS (aiTBS) to either the left or right DLPFC in participants with major depressive disorder in later life, and to assess for changes in connectivity within and between brain networks associated with antidepressant response, i.e., the “triple networks.” (37, 38) We hypothesized that aiTBS to the right DLPFC would be similar in antidepressant efficacy to left hemisphere treatment, and that connectivity changes between network nodes would also be similar between left and right hemisphere groups, reflecting a common network response to iTBS.

## 2 Materials and methods

This was an open-label, prospective cohort study of accelerated fMRI-targeted iTBS conducted in 44 patients aged 50 years and older with a diagnosis of major depressive disorder. This protocol was reviewed and approved by the UNM Health Sciences Center Human Research Review Committee (HRRC #19-531). Recruitment, testing, and stimulation methods, along with results in a subset of 25 participants receiving right DLPFC iTBS were previously reported (31). To compare the efficacy of left versus right DLPFC accelerated fMRI-guided iTBS, an additional group of 19 patients matched for age and sex was recruited and enrolled. Of these, 18 underwent left DLPFC iTBS using the identical protocol, while one participant underwent right DLPFC iTBS due to inability to tolerate left side iTBS due to trigeminal irritation, resulting in 26 total participants receiving right side and 18 receiving left side treatments. Sample size calculation using G*Power (Heinrich Heine Universität, Düsseldorf, Germany) determined that a total sample size of 40 participants would have 80% power (alpha = 0.05) to detect a small to medium difference between groups (interaction Cohen’s *f* = 0.2 (small to medium effect; η^2^) ~ 0.04).

### 2.1 Recruitment

Recruitment took place through the UNM Mental Health Center and associated clinics. All participants were referred for consideration of rTMS treatment for major depression, having not benefitted from various therapeutics such as oral antidepressants, psychotherapy, esketamine, electroconvulsive therapy (ECT), or once-daily rTMS. Participants were screened via phone and chart review for inclusion and exclusion criteria.

### 2.2 Inclusion/exclusion criteria

To be enrolled in the study, participants met the following inclusion criteria: 1) aged 50-79; 2) had received a diagnosis of major depressive disorder of at least six months’ duration preceding study entry, confirmed by two independent board-certified psychiatrists according to DSM-5 criteria; 3) had undertaken four or more adequate trials of antidepressants in the current episode; 4) scored of 10 or higher on the Quick Inventory of Depressive Symptomatology (16 item)(Self-Report)(QIDS-SR-16) at time of study entry; and 5) fluent in English. Exclusion criteria included: 1) history of seizure; 2) history of a major neurocognitive disorder or central nervous system disorder diagnosis; 3) implanted ferromagnetic material or contraindication to obtaining MRI; 4) pregnancy; 5) current incarceration; 6) inability to complete the protocol; 7) medical instability resulting in hospitalization or emergency department visit within the past month; 8) psychotropic medication change or treatment with neuromodulation or esketamine within one month preceding study entry.

### 2.3 Visit 1 assessment

After screening and consent, participants underwent demographic survey (age, sex, socioeconomic status, educational attainment, ethnicity, race, and handedness); assessment of depression history and treatment; mood and anxiety symptom assessment with the Inventory of Depressive Symptomatology for Clinicians-30 (IDS-C_30_, primary outcome measure); Generalized Anxiety Disorder-7 (GAD-7); Snaith-Hamilton Assessment of Pleasure Seeking for Clinician Administration (SHAPS-C); Temporal Experience of Pleasure Scale (TEPS); and the Behavioral Inhibition System/Behavioral Approach System Scale (BIS/BAS). These instruments were chosen in line with prior study protocols combining imaging and neuromodulation (39, 40).

### 2.4 MRI

At the baseline visit, participants underwent structural and resting-state functional magnetic resonance imaging (MRI) on a 3T Siemens Prisma scanner. High-resolution T_1_- and T_2_-weighted structural images and two runs of 6 minutes of resting-state functional MRI (rsfMRI) were obtained. For structural scans: repetition time (TR) = 2530 milliseconds (ms), echo time (TE) = 1.64, 3.5, 5.36, 7.22, 9.08 ms, inversion time (TI) = 1200 ms, flip angle = 7.0°, slices = 192, field of view = 256, matrix 256 x 256, voxel size = 1.0 x 1.0 x 1.0 millimeter (mm). For resting-state scans: TR = 480 ms (multiband acceleration factor of 8), TE = 29 ms, flip angle (FA) = 75°, slices = 192, voxel size = 2.0 × 2.0 × 2.0 mm. The T_1_ was preprocessed by parcellating with Freesurfer 6.0.0 and then aligned to rsfMRI data (41). The rsfMRI was preprocessed using AFNI’s recommended pipeline (example 11) afni_proc.py with AFNI 20.2.18 (42). The first four volumes of each run were dropped and each run was aligned and despiked, slice time corrected, distortion corrected, warped to Montreal Neurological Institute (MNI) space, blurred with a 4mm full width at half maximum (FWHM) Gaussian kernel, and scaled to a mean of 100. Nuisance signals were regressed and outlying volumes censored, and the runs were concatenated.

### 2.5 Targeting

Determination of neuronavigation targets was based on published methods of Ning et al. (43) A seed region was defined using the Brainnetome atlas region corresponding to the subgenual cingulate cortex (187, 188), and the bounding search region within the DLPFC in each hemisphere was created from the Brainnetome regions (15, 16, 19, 20, 21, 22) making up Brodmann areas 9 and 46 (44). Functional connectivity was measured by correlating time-series data from the pre-processed rsfMRI data for the seed region with each voxel in the search regions. A mask was created with the maximum anticorrelated voxel in the search region. Structural T1 images and the functional mask were then exported to a Localite neuronavigation system for registration during stimulation sessions.

### 2.6 Stimulation

Participants received a total of 45 sessions over two weeks (five sessions/day, nine weekdays) of iTBS to the cortical target with a MagPro X100 equipped with a Cool-B70 coil (Magventure Inc., Alpharetta, GA). Co-registration of the MRI data in the Localite neuronavigation system was performed with head landmarks at the nasion and bilateral tragus. The mask with the functional target was overlaid on the structural images and projected orthogonally to the nearest scalp surface for coil positioning. Coil rotation at the scalp projection was specified as 45° from midline, with the coil handle pointing posteriorly. Coil tilt was maintained tangential to the plumb line from scalp projection to brain target. Deviation from target during iTBS was monitored and the coil repositioned for any displacements greater than three millimeters. In each session, 1800 pulses were delivered in 60 trains of 10 triplet bursts (pulse frequency 50 Hz, burst frequency 5 Hz), 2 second train duration, 8 second intertrain interval in accordance with recently published accelerated iTBS protocols by Cole et al. (45) Pulses were delivered at 120% of resting motor threshold (RMT), defined as the minimum amount of energy to obtain five out of 10 motor evoked potentials with peak to peak amplitude of at least 50 uV in the abductor pollicis brevis muscle on electromyography, in accordance with parameters from the iTBS noninferiority study by Blumberger et al. (13) If patients could not tolerate 120% of RMT due to scalp discomfort, the highest tolerable stimulation intensity up to 120% RMT was delivered. Each iTBS session was separated by 50 minutes, based on prior work demonstrating this time frame as the optimal recovery time between sessions for an accelerated protocol (46).

### 2.7 Visit 2 and 3 assessments

After 15 sessions participants repeated all behavioral assessments (Visit 2). They then received 30 more sessions. If there was minimal improvement (<10%) noted in IDS-C_30_ score at Visit 2 or development of intolerable side effects, the participant was switched to stimulation of the opposite hemisphere for the remainder of treatment. The day following completion of the 45th session, participants repeated behavioral assessments and cognitive testing (Visit 3). At one month and three months following the protocol, the subjects were contacted by phone and assessed with the IDS-C_30_.

### 2.8 Statistical analysis

Means, standard deviations, and 95% confidence intervals (CI) for demographics and baseline behavioral and cognitive measures were calculated in SPSS Statistics 26 (IBM; Armonk, NY). Repeated-measures analyses of variance and effect sizes expressed as partial eta squared (ηp^2^) were calculated for behavioral outcomes using linear mixed models in R v. 4.1.3 (R Foundation; Vienna, Austria). Custom linear contrasts for change from baseline to Visit 2 and Visit 3 within Sides with 95% CI were calculated using SAS v9.4 (SAS Institute Inc., Cary, NC, USA). Analysis models used an intention to treat approach (ITT) with Visit, randomly assigned Side and Visit x Side interaction effects. Bonferroni correction for multiple comparisons was applied for main and interaction effects. Impact of switching sides after Visit 2 was explored in sensitivity analyses that added time-varying covariates to linear mixed models from above that coded for switchers after the Visit 2 measurement. One model estimated the effect of any switch and a second model estimated the effect of left to right and right to left switch separately.

### 2.9 Image analysis

Brainnetome regions for analysis were selected based on inclusion in prespecified networks involved in emotion regulation from the Yeo 7-network atlas (47): salience (ventral attention), central executive (frontoparietal), default mode, and limbic and subcortical (amygdala, hippocampus, basal ganglia) networks. Time courses were averaged within each Brainnetome region and connectivity matrices were calculated for each individual subject at each testing visit. Repeated-measures analysis of variance (rmANOVA) was calculated for each region-region connectivity pair within and between the selected networks. To determine whether stimulation side differences were present between networks, rmANOVAs were calculated for each network pair (4 x 4 network matrix) resulting in 10 unique network pairs. Change in connectivity between Visit 1 and Visit 2, Visit 2 and Visit 3, and Visit 1 and Visit 3 was calculated for each network and by side of stimulation. Induced electric field strength (*|E|*) was calculated based on the intensity of stimulation delivered (% of maximum device output) using Simulation of Non-Invasive Brain Stimulation (SimNIBS) ^68,69^ for each personalized target, as well as in each parcellated region across the whole brain using the Human Connectome Project multimodal atlas parcellation (48) (HCP-MMP) for each subject and then averaged for all participants in each group.

## 3 Results

### 3.1 Baseline characteristics

The left and right DLPFC stimulation groups were well-matched in terms of demographic and clinical characteristics, including age, sex, education level, body mass index, premorbid intelligence, baseline IDS-C30 score, presence of comorbid psychiatric diagnoses, history of treatment with ECT, and motor threshold. (See **Table 1**.) There was a trend toward a higher number of antidepressant trials in the left side group (11.8 vs 9.1, p = .06).

**Table 1:** Demographic and clinical characteristics of the study population.

Variable	Right side (N = 26)	Left side (N = 18)	*p* value
Age, years	63.9 ± 9.1 [60.2,67.6]	65.6 ± 5.2 [63.0,68.2]	.78
Sex			
Female/Male	23/3	13/5	.24
Education (years)	6.5 ± 1.6 [5.9,7.1]	6.3 ± 1.6 [5.5,7.1]	.51
BMI	29.2 ± 7.1 [26.3,32.1]	27.4 ± 5.7 [24.6,30.2]	.54
Ethnicity			
Non-Hispanic	24 (92)	14 (78)	.21
Hispanic	2 (8)	4 (22)	
Race			
Caucasian	24 (92)	17 (94)	1.00
Other	2 (8)	1 (6)	
Comorbid psychiatric diagnoses			
GAD	8(31)	9 (50)	1.00
PTSD	8 (31)	8 (44)	.53
Other	3 (12)	5 (28)	.24
History of ECT	8 (31)	4 (22)	.73
TOPF scaled score	111 ± 13.1 [105.7,116.3]	113 ± 11.5 [107.3,118.7]	.84
# Antidepressant trials	9.1 ± 5.9 [6.7,11.5]	11.8 ± 8.4 [7.6,16.0]	.06
Baseline IDS-30-C	38.9 ± 9.2 [35.2,42.6]	37.3 ± 8.9 [32.9,41.7]	.28
Mean motor threshold	36.7% ± 6.5 [34.1,39.3]	37.7% ± 9.3 [33.1,42.3]	.98
Mean treatment intensity	41.9% ± 6.9 [39.1,44.7]	45.2% ± 11.2 [39.6,50.8]	.44
# Switchers	6 (23)	3 (17)	.60

Values are number (%) or mean ± standard deviation and 95% CI [lower,upper]. P values represent significance calculated using Mann-Whitney U Tests for continuous variables, and Fisher’s Exact test for ordinal variables.

### 3.2 Symptom changes

**Figure 1:**
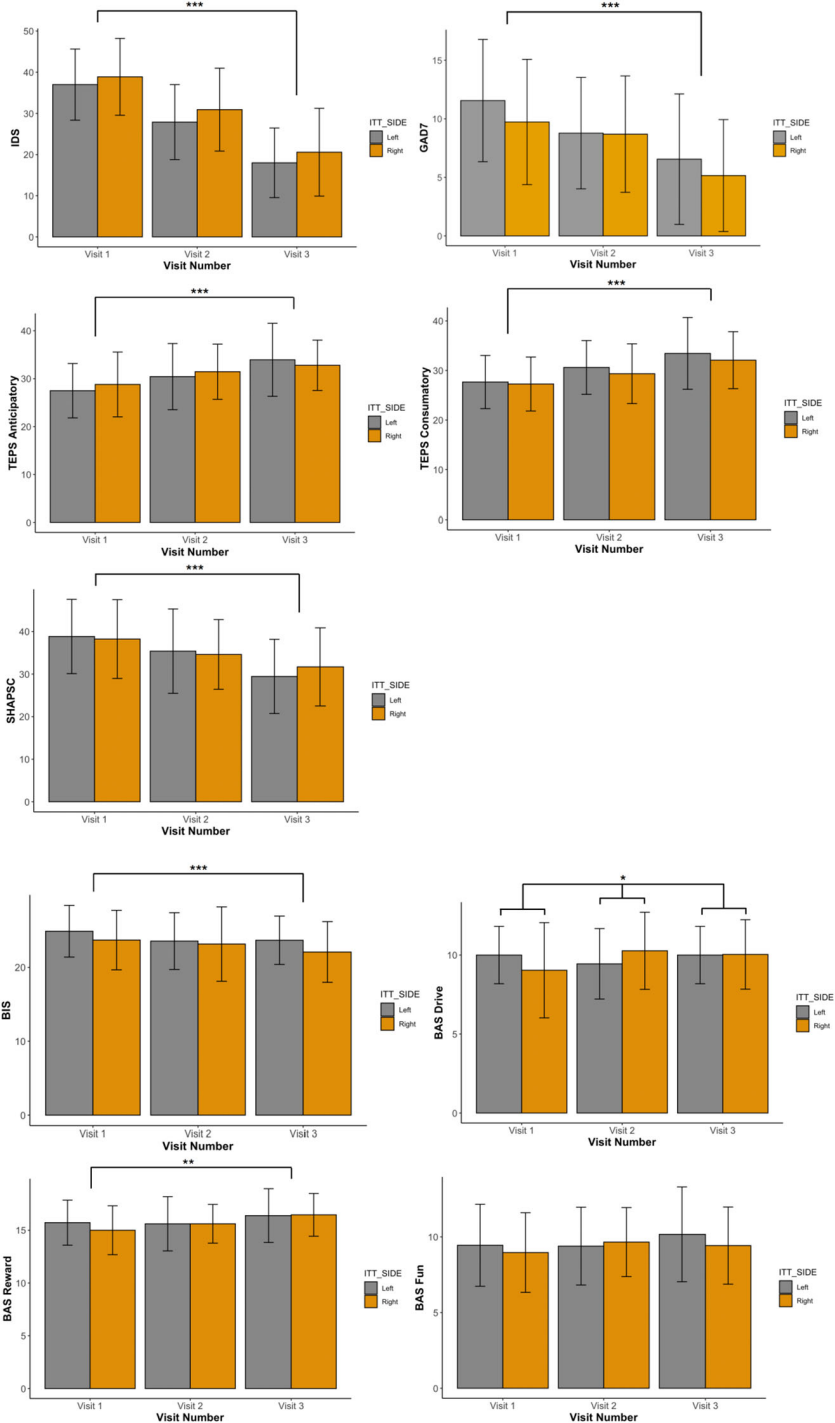
Bar graphs of means and standard deviations for measures of depression (IDS-C), generalized anxiety (GAD-7), anhedonia (SHAPS, TEPS), and approach/avoidance (BIS/BAS) in left and right stimulation groups for each visit. Orange =right; gray = left. Framing lines without bracket ends indicate significant VISIT effect; framing lines with bracket endsindicate significant VISIT x SIDE effect. * indicates p <. 05. ** indicates p =. 001. *** indicates p = .0001 or less.

**Figure 2:**
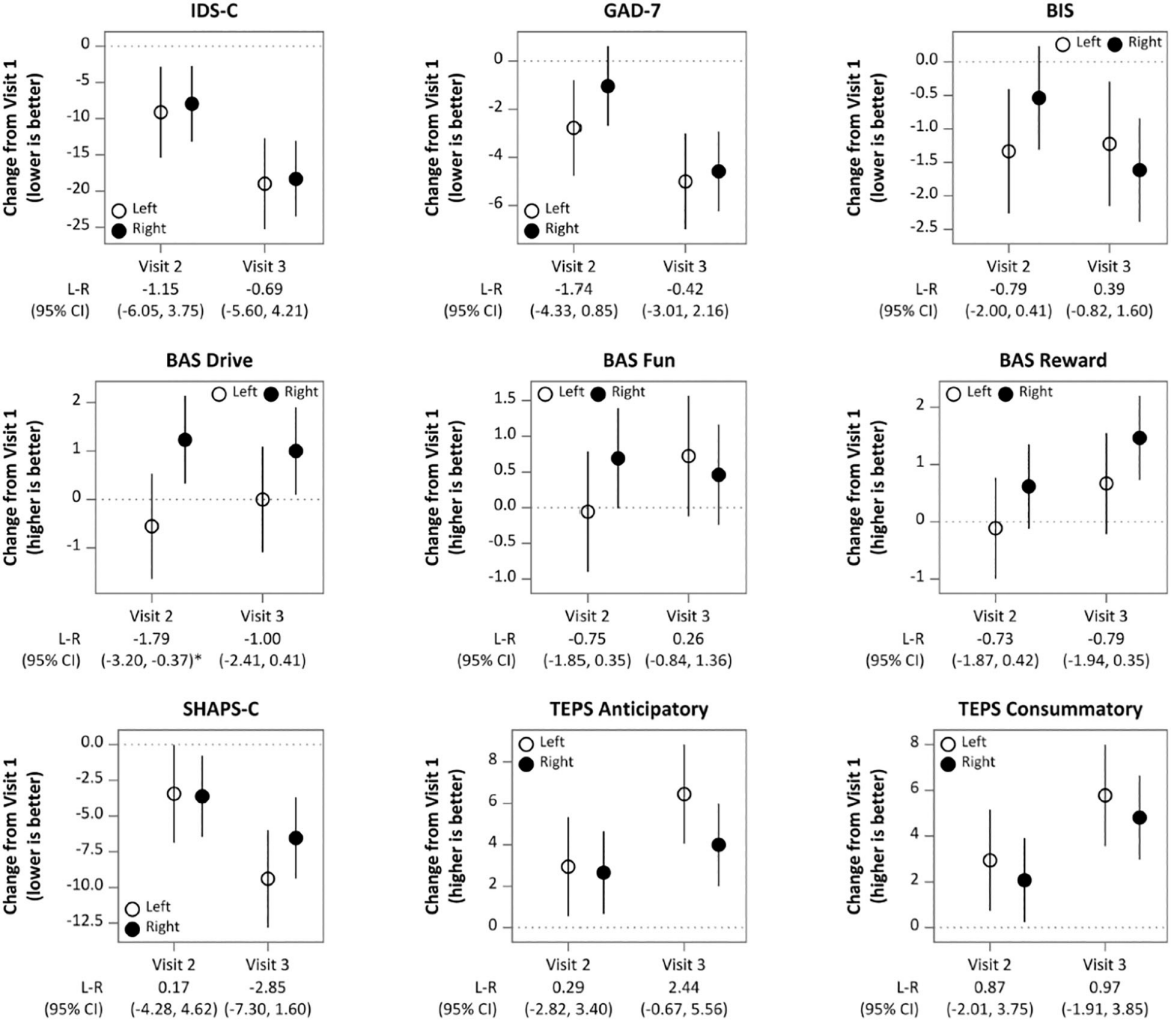
Change from Visit 1 and 95% CI for behavioral variables estimated by linear mixed model analyses (**Table 2**). L-R is left side change minus right side change at Visit 2 (after 15 sessions) and at Visit 3 (end of treatment). *p <. 05.

IDS-C30 scores improved significantly from Visit 1 to Visit 3 in both groups (left side IDS-C30 V1 = 37.0 ± 9.0, 95% CI [32.5,41.5]; V2 = 27.9 + 9.1 [23.4,32.4]; V3 = 18.0 ± 8.5 [13.8,22.2]; right side V1 = 38.9 ± 9.3 [35.1,42.7]; V2 = 31.0 ± 10.1 [26.9,35.1]; V3 = 20.6 ± 10.7 [16.3,24.9]; VISIT: F(2,84) = 114.714, p <.0001, p</sub>2 = 0.732). (See **Table 2**; **Figure 1**.) There was no significant main effect of initial side of stimulation (SIDE: F(1,42) = 0.959, p = .33, p</sub>2 = 0.022) nor was there an interaction effect observed: participants receiving right DLPFC stimulation demonstrated the same degree of improvement from Visit 1 to Visit 3 as the left DLPFC stimulation group (VISIT x SIDE: F(2,84) = 0.110, p = .90, p</sub>2 = 0.003). Significant improvements were also observed for both groups as a function of VISIT in the SHAPS-C (F(2,84) = 25.450, p <.0001, p</sub>2 = 0.377), TEPS Anticipatory (F(2,84) = 22.327, p <.0001, p</sub>2 = 0.347), TEPS Consummatory (F(2,84) = 26.748, p <.0001, p</sub>2 = 0.389), BIS (F(2,84) = 11.263, p = .0003, p</sub>2 = 0.211), BAS Reward (F(2,84) = 7.418, p = .001, p</sub>2 = 0.150) and GAD-7 (F(2,84) = 27.471, p <.0001, p</sub>2 = 0.395). No effect of SIDE and no interaction effect between VISIT and SIDE was detected for any behavioral variables, with the exception of BAS Drive, which demonstrated a small interaction effect (F(2,84) = 3.168, p = .047, p</sub>2 = 0.070) (see **Table 3**; **Figure 2**). After Bonferroni correction for multiple comparisons, this effect was no longer significant.

**Table 2:** Means, standard deviations, effect sizes, and p values for main effects and interaction effects for all behavioral variables.

Variable	Side	Visit 1 mean ± SD 95% CI [+/-]	Visit 2 mean ± SD 95% CI [+/-]	Visit 3 mean ± SD 95% CI [+/-]	Effect	F (dF)	p value	Effect size (ηp^2^)
IDS-C_30_	Left	37.0 ± 9.0	27.9 ± 9.1	18.0 ± 8.5	VISIT	114.714 (2,84)	<.0001**	0.732
		[32.5,41.5]	[23.4,32.4]	[13.8,22.2]				
	Right	38.9 ± 9.3	31.0 ± 10.1	20.6 ± 10.7	SIDE	0.959 (1,42)	.33	0.022
		[35.1,42.7]	[26.9,35.1]	[16.3,24.9]				
					VISIT x SIDE	0.110 (2,84)	.90	0.003
GAD7	Left	11.6 ± 5.2	8.8 ± 4.8	6.6 ± 5.6	VISIT	27.471 (1.68,70.74)	<.0001**	0.395
		[9.0,14.2]	[6.4,11.2]	[3.8,9.4]				
	Right	9.7 ± 5.3	8.7 ± 5.0	5.2 ± 4.8	SIDE	0.647 (1,42)	.14	0.015
		[7.6,11.8]	[6.7,10.7]	[3.3,7.1]				
					VISIT x SIDE	0.972 (1.68,70.74)	.23	0.023
BIS	Left	24.9 ± 3.5	23.6 ± 3.8	23.7 ± 3.3	VISIT	11.263 (2,84)	<.0001**	0.211
		[23.2,26.6]	[21.7,25.5]	[22.1,25.3]				
	Right	24.0 ± 4.0	23.2 ± 5.0	22.1 ± 4.1	SIDE	0.782 (1,42)	.38	0.018
		[22.4,25.6]	[21.2,25.2]	[20.4,23.8]				
					VISIT x SIDE	1.982 (2,84)	.14	0.045
BAS Drive	Left	10.0 ± 1.8	9.4 ± 2.2	10.0 ± 1.8	VISIT	1.029 (2,84)	.362	0.024
		[9.1,10.9]	[8.3,10.5]	[9.1,10.9]				
	Right	9.0 ± 3.0	10.3 ± 2.4	10.0 ± 2.2	SIDE	0.003 (1,42)	.96	<0.001
		[7.8,10.2]	[9.3,11.3]	[9.1,10.9]				
					VISIT x SIDE	3.168 (2,84)	.05	0.070
BAS Fun	Left	9.4 ± 2.7	9.4 ± 2.6	10.2 ± 3.1	VISIT	0.106 (2,84)	.11	0.052
		[8.1,10.7]	[8.1,10.7]	[8.7,11.7]				
	Right	9.0 ± 2.6	9.7 ± 2.3	9.4 ± 2.5	SIDE	0.188 (1,42)	.67	0.004
		[7.9,10.1]	[8.8,10.6]	[8.4,10.4]				
					VISIT x SIDE	1.799 (2,84)	.17	0.041
BAS Reward	Left	15.7 ± 2.1	15.6 ± 2.6	16.4 ± 2.5	VISIT	7.418 (2,84)	.001**	0.150
		[14.7,16.7]	[14.3,16.9]	[15.2,17.6]				
	Right	15.0 ± 2.3	15.6 ± 1.8	16.5 ± 2.0	SIDE	0.131 (1,42)	.72	0.003
		[14.1,15.9]	[14.9,16.3]	[15.7,17.3]				
					VISIT x SIDE	1.164 (2,84)	.32	0.027
SHAPS-C	Left	38.8 ± 8.7	35.4 ± 9.9	29.4 ± 8.7	VISIT	25.450 (2,84)	<.0001**	0.377
		[34.5,43.1]	[30.5,40.3]	[25.1,22.7]				
	Right	38.2 ± 9.3	34.6 ± 8.2	31.7 ± 9.2	SIDE	0.014 (1,42)	.91	0.0003
		[34.3,42.0]	[31.3,37.9]	[28.0,35.4]				
					VISIT x SIDE	1.151 (2,84)	.32	0.027
TEPS Anticipatory	Left	27.5 ± 5.7	30.4 ± 6.9	33.9 ± 7.6	VISIT	22.327 (2,84)	<.0001**	0.347
		[24.7,30.3]	[27.0,33.8]	[30.1,37.7]				
	Right	28.8 ± 6.8	31.4 ± 5.8	32.8 ± 5.3	SIDE	0.054 (1,42)	.82	0.001
		[26.1,31.5]	[29.1,33.7]	[30.7,34.9]				
					VISIT x SIDE	1.457 (2,84)	.24	0.034
TEPS Consummatory	Left	27.7 ± 5.4	30.6 ± 5.4	33.4 ± 7.2	VISIT	26.748 (2,84)	<.0001**	0.389
		[25.0,30.4]	[27.9,33.3]	[29.8,37.0]				
	Right	27.3 ± 5.4	29.3 ± 6.0	32.1 ± 5.7	SIDE	0.400 (1,42)	.53	0.009
		[25.1,29.5]	[26.9,31.7]	[29.8,34.4]				
					VISIT x SIDE	0.271 (2,84)	.76	0.006

Bolded values indicate statistically significant p-values.

**Table 3:** Change from Visit 1 summary for behavioral variables from linear mixed model analyses (**Table 2**).

		Visit 2 (after 15 sessions)		Visit 3 (end of treatment)		
Variable	Side	Change from Visit 1 (95% CI)	L-R Change (95% CI)	Change from Visit 1 (95% CI)	L-R Change (95% CI)	Omnibus p value
IDS-C_30_ (lower is better)	Left	-9.11 (-15.40, -2.82)**	-1.15 (-6.05, 3.75)	-19.00 (-25.29, -12.71)***	-0.69 (-5.60, 4.21)	.896
	Right	-7.96 (-13.19, -2.73)**		-18.31 (-23.54, -13.08)***		
GAD-7 (lower is better)	Left	-2.78 (-4.77, -0.79)**	-1.74 (-4.33, 0.85)	-5.00 (-6.99, -3.01)***	-0.42 (-3.01, 2.16)	.382
	Right	-1.04 (-2.69, 0.62)		-4.58 (-6.23, -2.92)***		
BAS Drive (higher is better)	Left	-0.56 (-1.64, 0.53)	-1.79 (-3.20, -0.37)*	0.00 (-1.09, 1.09)	-1.00 (-2.41, 0.41)	.047
	Right	1.23 (0.33, 2.14)**		1.00 (0.10, 1.90)*		
BAS Fun (higher is better)	Left	-0.06 (-0.90, 0.79)	-0.75 (-1.85, 0.35)	0.72 (-0.12, 1.57)+	0.26 (-0.84, 1.36)	.172
	Right	0.69 (-0.01, 1.39)+		0.46 (-0.24, 1.16)		
BAS Reward (higher is better)	Left	-0.11 (-0.99, 0.77)	-0.73 (-1.87, 0.42)	0.67 (-0.22, 1.55)	-0.79 (-1.94, 0.35)	.317
	Right	0.62 (-0.12, 1.35)+		1.46 (0.73, 2.20)***		
BIS (lower is better)	Left	-1.33 (-2.26, -0.40)**	-0.79 (-2.00, 0.41)	-1.22 (-2.15, -0.29)*	0.39 (-0.82, 1.60)	.144
	Right	-0.54 (-1.31, 0.23)		-1.62 (-2.39, -0.84)***		
SHAPS-C (lower is better)	Left	-3.44 (-6.86, -0.02)*	0.17 (-4.28, 4.62)	-9.39 (-12.81, -5.97)***	-2.85 (-7.30, 1.60)	.321
	Right	-3.62 (-6.46, -0.77)*		-6.54 (-9.38, -3.69)***		
TEPS Anticipatory (higher is better)	Left	2.94 (0.55, 5.34)*	0.29 (-2.82, 3.40)	6.44 (4.05, 8.84)***	2.44 (-0.67, 5.56)	.239
	Right	2.65 (0.66, 4.64)**		4.00 (2.01, 5.99)***		
TEPS Consummatory (higher is better)	Left	2.94 (0.73, 5.16)**	0.87 (-2.01, 3.75)	5.78 (3.56, 7.99)***	0.97 (-1.91, 3.85)	.763
	Right	2.08 (0.24, 3.92)*		4.81 (2.97, 6.65)***		

L-R is the left side change minus the right side change at Visit 2 (after 15 sessions) and at Visit 3 (end of treatment). Omnibus p value is the Visit x Side interaction test. *** p<.001 ** p<.01 * p<.05 + p<.10.

### 3.3 Effects of switching

Six participants switched from right side to left side (R→L) after Visit 2, while three participants switched from left side to right side (L→R). →erent from nonswitchers on scores from Visit 2 to Visit 3 except for TEPS Consummatory where the L→R change was -5.24 (SE = 2.45, p = .036) compared to nonswitchers. R→L change over the same period was 1.98 (SE = 1.81, p = .30) compared to non-switchers. After correcting for multiple comparisons, the L→R difference was no longer significant.

### 3.4 Target and electric field distribution

**Figure 3:**
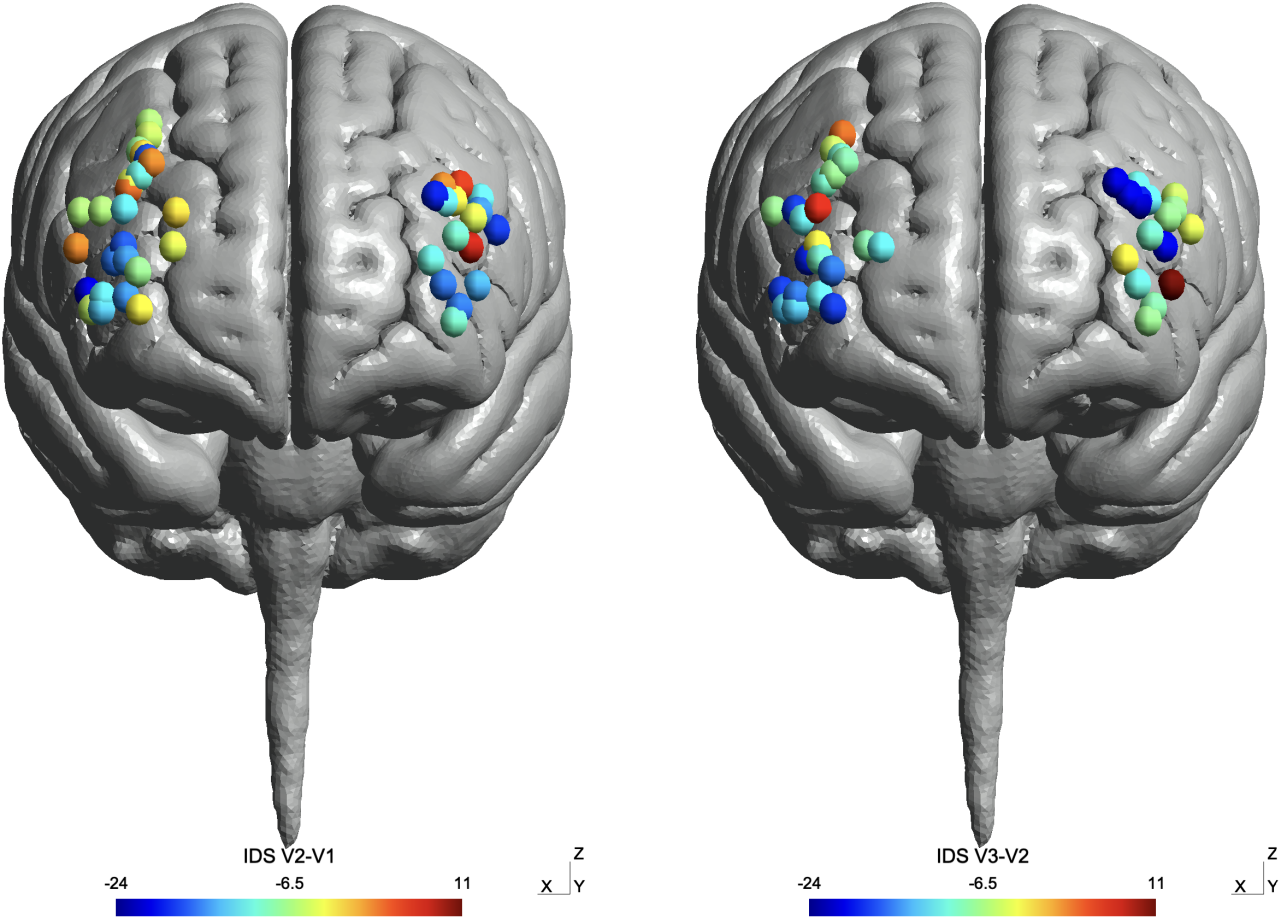
Target distribution across right and left DLPFC. Color coding of targets indicates change in IDS-C-30 score from V1 to V2 (left), and V1 to V3 (right) (cooler colors = greater improvement in depression symptoms).

**Figure 4:**
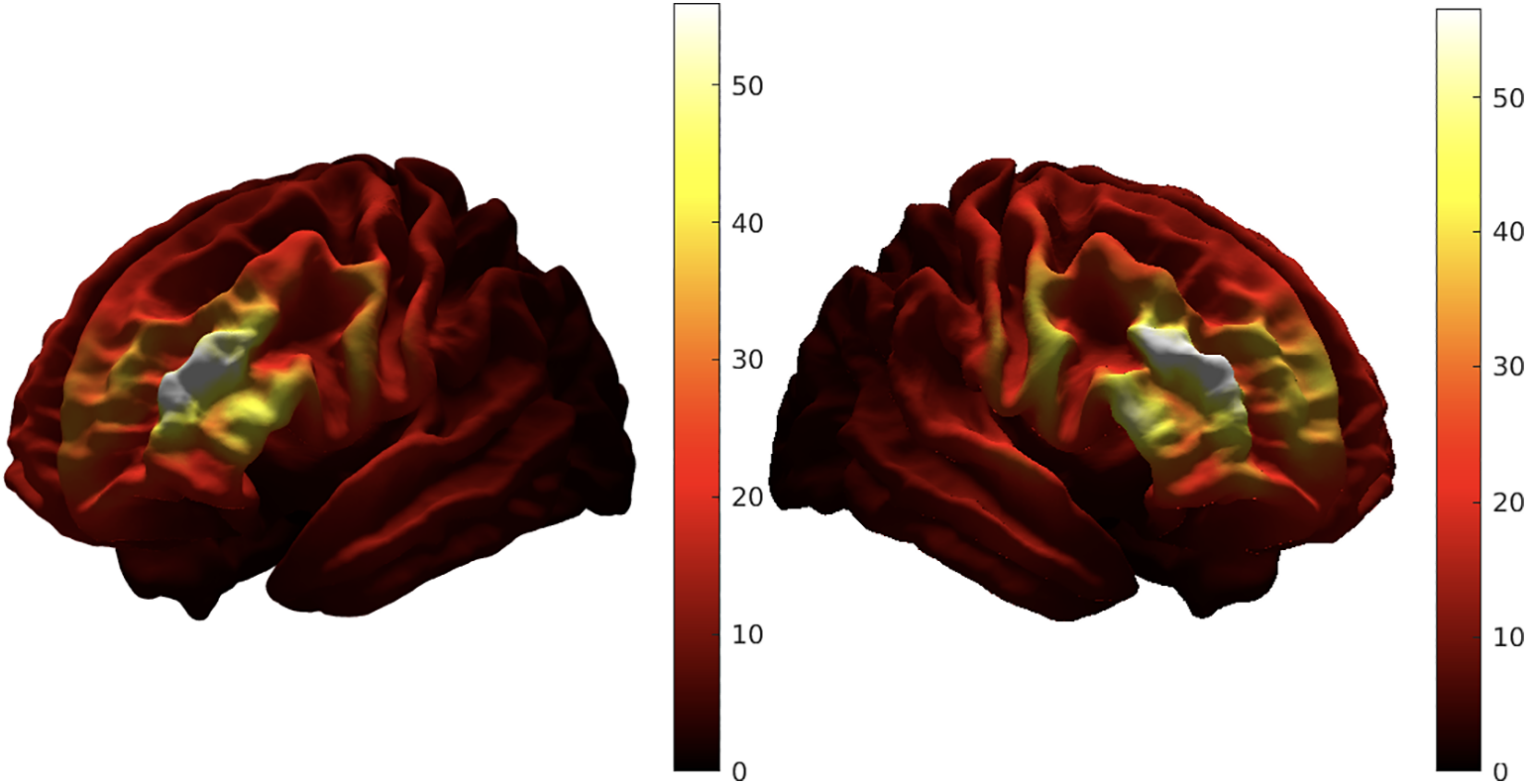
Average induced electric field strength of stimulation (|E|; V/m) for left and right DLPFC stimulation groups.

Targets were distributed over a broad area in the right DLPFC, but were more tightly clustered in the middle frontal gyrus in the left DLPFC group (see **Figure 3**). By end of treatment, more efficacious targets in the right DLPFC stimulation group tended to occur anteriorly and inferiorly within the region. Effective targets in the left DLPFC were located more posteriorly and superiorly within the bounding region. Induced electric field intensity (|E|) was comparable between the stimulation groups, with maximal |E| occurring within the middle frontal gyrus in both stimulation groups (see **Figure 4**); due to the broader spatial distribution of targets with the right hemisphere, nonnegligible levels of |E| (> 30 V/m) were delivered to the superior frontal gyrus, inferior frontal gyrus, and precentral gyrus. As was previously reported in Quinn et al, 2023,33 |E| occurring in a subregion of the inferior frontal gyrus (Brodmann area 47) was moderately correlated with antidepressant effect in the patients receiving right side stimulation. However, there was no significant correlation between |E| in any DLPFC subregion and antidepressant response in the left side group.

### 3.5 Connectivity changes

**Figure 5:**
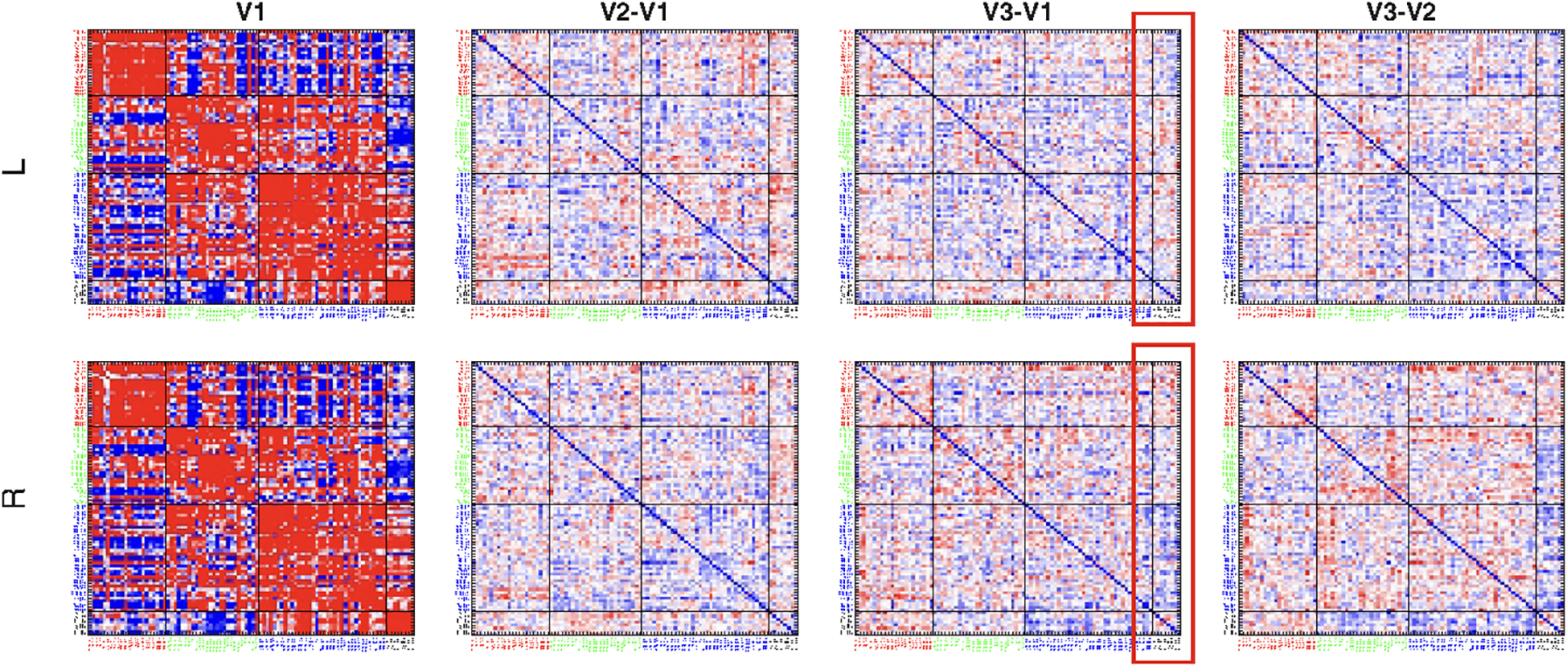
Connectivity values between nodes of the default mode (blue labels), salience (red labels), limbic (black labels), and central executive networks (green labels) at Visit 1 (V1) and change in connectivity pairs between Visit 1 and 2 (V2-V1), 2 and 3 (V3-V2), and 1 and 3 (V3-V1) for left (L) and right (R) hemisphere groups. Cooler colors (i.e., blue) indicate anticorrelated activity at V1, or decrease in connectivity between visits; warmer colors (i.e., red) indicate correlated activity at V1, or increase in connectivity between visits. Region groupings by network domain indicated by colored labels and bolded black lines. Red box indicates limbic network domain connectivity change in R side group from V1 to V3.

Baseline connectivity values between regions of the default mode, salience, central executive, and limbic networks were not significantly different between left and right DLPFC groups (see **Figure 5**). Broad changes in connectivity were observed from Visit 1 to Visit 3 in multiple ROI-ROI pairs in both groups, particularly between regions in the limbic network and regions in the other three networks (see **Supplementary Tables 1**, **2**). However, these changes were not significant after correction for false discovery rate (all p >.2). At the network domain level, there were significant changes in network-network connectivity observed from Visit 1 to Visit 3 in the right DLPFC group; these predominantly reflected a decrease in connectivity between the limbic network and the triple networks between Visit 2 and Visit 3 (see **Table 4**). The left side group demonstrated only limited changes in limbic network domain connectivity; therefore a significant difference between right and left DLPFC groups in default mode-limbic connectivity change from Visit 1 to Visit 3 was observed (F(1,41) = 4.311; p = .04; p</sub>2 = .095). After correction for false discovery rate, changes in network connectivity were not found to correlate significantly with change in depression scores (all p >.6). (See **Figure 5**).

**Table 4:** Significant changes in network domain connectivity in left versus right side stimulation groups.

Side	Network domain pair	Visit interval	F (dF)	p value	Effect size (p^2^)
Left	Limbic-Limbic	V2-V3	6.397 (1,17)	.02	.273
Right	Default Mode-Limbic	V1-V3	8.268 (1,21)	.009	.282
Right	Default Mode-Limbic	V2-V3	4.371 (1,21)	.05	.172
Right	Salience-Limbic	V2-V3	8.887 (1,21)	.007	.297
Right	Central Executive-Limbic	V2-V3	9.335 (1,21)	.006	.308
Left vs Right	Default Mode-Limbic	V1-V3	4.311 (1,41)	.04	.095

## 4 Discussion

### 4.1 Effect of left and right DLPFC iTBS on depression, anhedonia, and anxiety symptoms

Behavioral effects of accelerated iTBS to the left versus right DLPFC were not significantly different, with both approaches demonstrating large reductions in depression on the IDS-C_30_ and anhedonia (TEPS, SHAPS-C). While we are not able to exclude the possibility that non-stimulation factors are the basis of the behavioral effects, these findings align with prior studies that iTBS to the right DLPFC may be an effective alternative to left DLPFC iTBS for late-life depression, both in accelerated (31, 32) as well as once-daily formats (32). Our study also demonstrates that left and right side iTBS may have similar effects on a measure of generalized anxiety (GAD-7) and approach/avoidance behavior (BIS/BAS). Multiple prior studies have demonstrated that anxiety symptoms respond favorably to inhibitory rTMS protocols to the right hemisphere (49), and were based on a hypothesis of asymmetric distribution of anxiety circuitry between the hemispheres. Anxiety symptoms are frequently comorbid with major depression, and may originate from dysfunction in overlapping brain networks (50, 51), therefore a concomitant decrease in anxiety as depression improves is not surprising. However, our study suggests that anxiety circuitry may be more generally symmetric than previously thought, and an iTBS protocol to either hemisphere can have anxiolytic effects, offering hope for patients who are not able to receive or tolerate rTMS to a particular hemisphere.

### 4.2 Hemispheric preference for iTBS

Owing to the design of our protocol, which permitted switching to the contralateral hemisphere after fifteen sessions, we observed a minority of participants in both groups (17% L→R; 23% R→L) who did not demonstrate an initial response from stimulation. After switching, several of these participants demonstrated significant benefit on the contralateral side, suggesting that there may be an individual hemispheric preference for iTBS for depression. We believe our study is the first to explore this phenomenon of hemispheric preference using an identical protocol (i.e., iTBS) in either hemisphere, as prior studies of switching used inhibitory protocols (e.g., 1Hz or cTBS) in the right hemisphere, and could not disambiguate preference for hemisphere from preference for stimulation type. Although the numbers of switchers in each group were small, our study did not demonstrate a clear superiority of one hemisphere over the other in terms of participants not responding to aiTBS and switching. It is not yet clear what factors may influence or predict which hemisphere will respond to stimulation in a given patient. Consistent with other case series (52), we have observed in our clinic that conventional left DLPFC iTBS can induce agitation or worsening of depression, and in those cases delivering iTBS to the contralateral hemisphere often results in better tolerability and clinical response. Several candidate mechanisms may explain this finding of hemispheric preference: 1) depression-related cortical areas may be asymmetric or larger in one hemisphere in a given person, making target engagement more likely; 2) even if bilaterally represented, depression circuitry may be more “receptive” to neuromodulation on a given side, e.g., the DLPFC on one side may have a greater structural connectivity, as has been suggested in diffusion tensor imaging studies (53); 3) the chosen target in switchers may simply have been inaccurate, and had a different target been selected a better result may have been obtained. Increased study of these patients who fail to respond to initial stimulation and benefit from switching hemispheres is needed to elucidate potential explanatory mechanisms.

### 4.3 Asymmetry of connectivity change and target distribution

Although left DLPFC and right DLPFC groups demonstrated nearly identical rates of response in depressive symptoms, connectivity changes between network domains observed in each group were asymmetric. Following stimulation, the right DLPFC group demonstrated significant reductions in connectivity between limbic and default mode network domains, whereas the left side group did not exhibit significant change. These changes appeared to be dose-dependent, with connectivity changes from Visit 1 to 2 generally less robust than from Visit 2 to 3. This finding runs counter to our initial hypothesis, in which we expected use of an identical stimulation protocol (iTBS) in both groups to lead to equivalent changes in network connectivity. The meaning of this difference in connectivity change between the groups is not clear, given the lack of correlation between connectivity change and antidepressant response in this cohort (38). Without a control condition, it is not possible to determine whether the observed changes are due to the intervention versus nonspecific study effects, such as expectation or staff interactions. The late-life age range of the cohort may have contributed to attenuation of connectivity-related phenomena generally, as older age is associated with reduction of connectivity across the brain (54). One possible explanation for asymmetric connectivity responses may be the mild asymmetry of target locations and |E| distribution between the hemispheres. Average |E| was similar between the right and left DLPFC groups despite the use of individualized targeting and dosing methods. However, in the right hemisphere targets were more broadly distributed over the DLPFC, resulting in higher average |E| in the inferior frontal gyrus, which is known to exert functional influences on the amygdala (55), than in the left hemisphere. A second possibility is that prefrontal network connectivity change may still be a lateralized functional response to iTBS, and not a consistent physiologic effect such as cortical excitability. This intepretation is supported by the fact that TMS-induced connectivity changes that correlate with antidepressant response are often observed in non-stimulated networks, rather than in the targeted region itself, i.e., the DLPFC (38). In one of the few imaging studies of identical stimulation delivered to both hemispheres, Schluter et al. showed that in a sample of 45 healthy individuals, the application of high frequency rTMS to the right DLPFC resulted in increased connectivity within the salience network, whereas the same protocol delivered to the left DLPFC resulted in diminished connectivity (56). In a recent preprint by Sun et al., a novel network in the right DLPFC that is positively correlated with the subgenual cingulate cortex was described, and TMS delivered to scalp location F3 (left DLPFC) versus F4 (right DLPFC) yielded different proportions of these prefrontal networks stimulated (57). Our asymmetric connectivity findings suggest that there may still be fundamental differences in network functions and topography between the hemispheres that are coming to light as more study protocols apply iTBS and excitatory stimulation to the right DLPFC.

### 4.4 Limitations

There are several limitations of this study that influence the interpretation of the above findings. Small sample sizes in each group reduce statistical power particularly in the analysis of functional connectivity, and the study is underpowered to detect a difference between two active treatments. The uneven numbers of participants in the groups may have negatively impacted statistical power to detect differences in effects, although the ratio of participants (60/40) Lack of a sham condition prevents inferences of causality with regard to iTBS and behavioral and clinical outcomes. Nonspecific effects such as staff interactions, behavioral activation from daily visits, or expectation could have contributed to the clinical effects observed in both groups. Likewise, the connectivity changes observed following treatment and differences between the groups cannot be ascribed definitively to the intervention. A larger study with sham conditions for stimulation to both hemispheres would permit isolation of the connectivity effects of left and right DLPFC iTBS and comparison of their efficacy rates. Finally, while this study utilized individualized connectivity-based targeting, it is not possible to discern the specific contribution of this paradigm to the overall treatment effect in each group. Fixing stimulation target at a conventional scalp target such as F3/F4 could have reduced variability in target distribution across groups.

## 5 Conclusion

In this small comparative efficacy study, accelerated iTBS to either the left or right DLFPC was effective in reducing symptoms of major depressive disorder, anhedonia, and generalized anxiety in a late-life depression population. There were no significant differences between left and right aiTBS in terms of effect sizes or rates of switching to the contralateral side. Connectivity changes with treatment were different between the two groups, with right hemisphere stimulation associated with decreased connectivity between the default mode and limbic network domains. Importantly, this study provides initial data comparing accelerated iTBS efficacy rates with an fMRI-guidance paradigm applied to the left and right DLPFC, and generates additional hypotheses about mechanisms by which iTBS may interact with emotion regulation network nodes in each hemisphere. Accelerated iTBS to the right DLPFC may be considered as a viable treatment option in addition to left DLPFC aiTBS. Larger studies are needed to confirm this finding and to better understand the mechanism of the antidepressant effect of iTBS in both hemispheres.

## Data Availability

The raw data supporting the conclusions of this article will be made available by the authors, without undue reservation.
