# Supplementary material for: Equivalent efficacy of left versus right hemisphere accelerated intermittent theta burst stimulation for major depressive disorder
Source: Front Psychiatry. 2026 Mar 11;17:1745388. doi: 10.3389/fpsyt.2026.1745388 (PMC13014255; doi:10.3389/fpsyt.2026.1745388)
Supplement: Supplementary file 1 [file DataSheet1.pdf]

Supplemental Table 1. Left DLPFC Stimulation group ROI-ROI significant connectivity changes and correlation with IDS-C_{30}.

Region1	Region2	Network1	Network2	Connectivity t-stat	IDS-C_{30} Correlation	P_uncorrected	P_fdr
MTG_L_4_1	Hipp_R_2_1	Default	Limbic	-0.6567648	-0.6891348	0.00110012	0.26014392
OrG_R_6_3	Hipp_L_2_1	Central executive	Limbic	0.2300549	-0.6712554	0.0016517	0.26014392
IFG_R_6_4	Amyg_L_2_1	Central executive	Limbic	-1.989957	-0.6660645	0.00184938	0.26014392
Amyg_L_2_1	Amyg_R_2_1	Limbic	Limbic	0.76402832	-0.6626983	0.00198781	0.26014392
MTG_L_4_4	Hipp_R_2_1	Default	Limbic	0.01179992	-0.6545623	0.00235831	0.26014392
MTG_R_4_1	Amyg_R_2_2	Central executive	Limbic	1.51585521	-0.6401558	0.00315457	0.28998322
SFG_L_7_1	Amyg_R_2_2	Central executive	Limbic	-0.8242473	0.6131395	0.00524584	0.37580032
PrG_R_6_5	Amyg_L_2_2	Salience	Limbic	0.78830996	0.61100183	0.00545084	0.37580032
IFG_L_6_6	Hipp_R_2_2	Salience	Limbic	0.07532513	0.59432866	0.0072855	0.4114426
pSTS_L_2_2	Hipp_R_2_2	Salience	Limbic	0.0094307	0.5889345	0.00797652	0.4114426
CG_L_7_4	Amyg_L_2_2	Default	Limbic	0.26488628	0.58702591	0.00823339	0.4114426
CG_R_7_7	Amyg_R_2_1	Default	Limbic	0.89726306	-0.5788176	0.00941582	0.4114426
CG_R_7_6	Amyg_L_2_1	Salience	Limbic	-0.9436413	-0.5769848	0.00969771	0.4114426
SFG_R_7_1	Hipp_R_2_2	Salience	Limbic	0.45521451	0.5675907	0.01125173	0.41959003
SFG_R_7_1	Amyg_R_2_2	Salience	Limbic	-1.6372146	0.56550758	0.0116221	0.41959003
OrG_R_6_2	Hipp_R_2_2	Default	Limbic	-0.0811193	-0.5625111	0.012172	0.41959003
INS_L_6_6	Amyg_L_2_1	Salience	Limbic	0.45569446	-0.5506278	0.01456225	0.44671182
IPL_L_6_4	Amyg_R_2_2	Default	Limbic	-0.4202623	0.55055195	0.01457863	0.44671182
MFG_L_7_1	Amyg_R_2_2	Salience	Limbic	-0.5975827	0.53360894	0.0186242	0.54063892
OrG_R_6_6	Amyg_R_2_1	Default	Limbic	-1.3283237	-0.5291819	0.0198158	0.54646822
INS_L_6_6	Amyg_L_2_2	Salience	Limbic	0.3498866	-0.5095327	0.02585233	0.59178751
PCL_L_2_1	Hipp_R_2_2	Salience	Limbic	0.43918279	0.4946413	0.03132066	0.59178751
PCL_L_2_1	Amyg_L_2_1	Salience	Limbic	1.18977671	0.48814675	0.03397026	0.59178751
MFG_L_7_5	Amyg_R_2_2	Default	Limbic	0.83915784	0.48588261	0.03493374	0.59178751
CG_R_7_3	Hipp_L_2_1	Salience	Limbic	0.17409601	-0.4854408	0.0351242	0.59178751
SFG_L_7_7	Hipp_L_2_2	Default	Limbic	-0.9692786	-0.4828521	0.0362563	0.59178751
PrG_R_6_5	Amyg_R_2_1	Salience	Limbic	1.05197941	0.48219212	0.03654941	0.59178751
MFG_L_7_4	Hipp_R_2_1	Central executive	Limbic	-1.4965718	0.4806609	0.03723648	0.59178751
ITG_R_7_6	Amyg_R_2_2	Central executive	Limbic	0.93025689	-0.4803405	0.03738151	0.59178751
OrG_L_6_1	Amyg_L_2_1	Default	Limbic	0.39842334	0.47011326	0.04224412	0.59178751
SFG_R_7_2	Amyg_L_2_2	Central executive	Limbic	-1.3762872	-0.4699561	0.04232244	0.59178751
STG_L_6_6	Hipp_L_2_1	Default	Limbic	0.24156966	0.46511554	0.04479015	0.59178751
OrG_R_6_1	Hipp_L_2_2	Default	Limbic	-0.9360261	-0.4637954	0.04548187	0.59178751
PrG_R_6_5	Hipp_R_2_1	Salience	Limbic	0.2064885	0.46339769	0.04569187	0.59178751
PrG_R_6_5	Amyg_R_2_2	Salience	Limbic	0.28867989	0.46053518	0.04722521	0.59178751
OrG_R_6_3	Hipp_R_2_2	Central executive	Limbic	0.43730246	-0.4593854	0.04785202	0.59178751

Supplemental Table 2. Right DLPFC Stimulation group ROI-ROI significant connectivity changes and correlation with IDS-C_{30}.

Region1	Region2	Network1	Network2	Connectivity_ Tstat	IDS-C Correlation	P_uncorrected	P_fdr
MTG_R_4_4	Hipp_L_2_2	Default	Limbic	-0.239879	-0.5600977	0.00544466	0.98655161
pSTS_R_2_2	Hipp_R_2_2	Salience	Limbic	1.95565862	-0.5537583	0.00611783	0.98655161
STG_R_6_6	Hipp_R_2_2	Default	Limbic	-0.6983827	-0.5333893	0.00876707	0.98655161
MFG_L_7_2	Hipp_L_2_1	Central executive	Limbic	-1.626437	0.52852885	0.00952264	0.98655161
MFG_R_7_7	Amyg_L_2_1	Central executive	Limbic	-1.1155192	-0.5266433	0.00982986	0.98655161
PrG_L_6_5	Hipp_L_2_1	Salience	Limbic	0.17031887	-0.4939288	0.0166002	0.98655161
CG_L_7_6	Hipp_R_2_2	Salience	Limbic	-0.6248088	0.47392712	0.02233966	0.98655161
IPL_L_6_4	Hipp_L_2_1	Default	Limbic	-1.2038251	0.4724265	0.02282763	0.98655161
OrG_L_6_6	Amyg_L_2_2	Default	Limbic	-1.3294432	0.47083134	0.0233557	0.98655161
Hipp_L_2_2	Hipp_R_2_1	Limbic	Limbic	0.35195779	0.47024991	0.02355061	0.98655161
Amyg_L_2_1	Hipp_L_2_2	Limbic	Limbic	0.87364841	0.45979905	0.02728232	0.98655161
CG_R_7_7	Amyg_L_2_2	Default	Limbic	-2.2646145	-0.4529683	0.02996654	0.98655161
CG_L_7_6	Hipp_R_2_1	Salience	Limbic	-0.6488045	0.45054373	0.03096832	0.98655161
STG_R_6_6	Hipp_L_2_1	Default	Limbic	-1.0359231	-0.4486319	0.03177683	0.98655161
ITG_L_7_4	Hipp_R_2_2	Default	Limbic	-0.7972811	-0.4425578	0.03445733	0.98655161
Hipp_L_2_1	Hipp_L_2_2	Limbic	Limbic	1.63626037	0.44160126	0.03489529	0.98655161
MTG_R_4_4	Hipp_R_2_2	Default	Limbic	-2.194213	-0.4404177	0.03544325	0.98655161
STG_L_6_6	Hipp_R_2_2	Default	Limbic	0.12875933	-0.4393147	0.03595999	0.98655161
Amyg_R_2_1	Hipp_R_2_1	Limbic	Limbic	0.02873905	0.43685182	0.03713536	0.98655161
INS_L_6_3	Hipp_L_2_1	Salience	Limbic	1.57822772	-0.4368272	0.03714727	0.98655161
IFG_L_6_5	Hipp_L_2_1	Salience	Limbic	1.06679472	-0.4366304	0.03724249	0.98655161
IPL_R_6_4	Amyg_L_2_1	Central executive	Limbic	-0.0772846	-0.4345862	0.03824312	0.98655161
SFG_R_7_3	Amyg_L_2_1	Default	Limbic	-2.2381593	-0.4329458	0.03906128	0.98655161
IPL_L_6_2	Hipp_R_2_2	Central executive	Limbic	-1.2081598	0.43239819	0.03933744	0.98655161
OrG_R_6_3	Hipp_R_2_1	Central executive	Limbic	0.73558724	-0.4312422	0.03992544	0.98655161
MFG_R_7_1	Hipp_L_2_1	Central executive	Limbic	-1.0898076	-0.4311042	0.03999612	0.98655161
STG_L_6_6	Hipp_L_2_1	Default	Limbic	-0.4814747	-0.4249784	0.04323273	0.98655161
IFG_L_6_4	Amyg_L_2_1	Default	Limbic	-1.8306811	0.42192974	0.04491806	0.98655161
OrG_L_6_2	Hipp_L_2_1	Default	Limbic	-0.6448587	-0.4217912	0.04499582	0.98655161
CG_L_7_6	Hipp_L_2_1	Salience	Limbic	-0.2747825	0.41434645	0.04933292	0.98655161
IPL_R_6_5	Amyg_R_2_2	Default	Limbic	0.64746019	-0.4135789	0.04979791	0.98655161
